# Automated ‘oscillometric’ blood pressure measuring devices: how they work and what they measure

**DOI:** 10.1038/s41371-022-00693-x

**Published:** 2022-05-30

**Authors:** James E. Sharman, Isabella Tan, George S. Stergiou, Carolina Lombardi, Francesca Saladini, Mark Butlin, Raj Padwal, Kei Asayama, Alberto Avolio, Tammy M. Brady, Alan Murray, Gianfranco Parati

**Affiliations:** 1 Menzies Institute for Medical Research, University of Tasmania, Hobart, TAS Australia; 2 Faculty of Medicine, Health and Human Sciences, Macquarie Medical School, Macquarie University, Sydney, NSW Australia; 3 The George Institute for Global Health, Sydney, NSW Australia; 4 Hypertension Center STRIDE-7, Third Department of Medicine, School of Medicine, National and Kapodistrian University of Athens, Sotiria Hospital, Athens, Greece; 5 Istituto Auxologico Italiano, IRCCS, Sleep Disorders Center & Department of Cardiovascular, Neural and Metabolic Sciences, San Luca Hospital, Milan, Italy; 6 Department of Medicine and Surgery, University of Milano-Bicocca, Milan, Italy; 7 Cardiology Unit, Cittadella Town Hopsital, Padova, Italy; 8 Department of Medicine, University of Alberta, Edmonton, AB Canada; 9 Department of Hygiene and Public Health, Teikyo University School of Medicine, Tokyo, Japan; 10 Studies Coordinating Centre, Research Unit Hypertension and Cardiovascular Epidemiology, KU Leuven Department of Cardiovascular Sciences, University of Leuven, Leuven, Belgium; 11 Division of Pediatric Nephrology, Johns Hopkins University School of Medicine, Baltimore, MD USA; 12 Faculty of Medical Sciences, School of Engineering, and Cardiovascular Physics, Newcastle University, Newcastle upon Tyne, UK

**Keywords:** Risk factors, Hypertension, Diagnosis

## Abstract

Automated ‘oscillometric’ blood pressure (BP) measuring devices (BPMDs) were developed in the 1970s to replace manual auscultatory BP measurement by mercury sphygmomanometer. Automated BPMDs that have passed accuracy testing versus a reference auscultatory sphygmomanometer using a scientifically accepted validation protocol are recommended for clinical use globally. Currently, there are many thousands of unique automated BPMDs manufactured by hundreds of companies, with each device using proprietary algorithms to estimate BP and using a method of operation that is largely unchanged since inception. Validated automated BPMDs provide similar BP values to those recorded using manual auscultation albeit with potential sources of error mostly associated with using empirical algorithms to derive BP from waveform pulsations. Much of the work to derive contemporary BP thresholds and treatment targets used to manage cardiovascular disease risk was obtained using automated BPMDs. While there is room for future refinement to improve accuracy for better individual risk stratification, validated BPMDs remain the recommended standard for office and out-of-office BP measurement to be used in hypertension diagnosis and management worldwide.

## Introduction

Manual auscultatory measurement of upper arm blood pressure (BP) with a mercury sphygmomanometer was the gold standard non-invasive test and mainstay clinical method to diagnose hypertension in the twentieth century [1, 2] This indirect measurement method, as well as automated BP methods, was used in the ground-breaking epidemiological and clinical trials that discovered the importance of high BP as a cardiovascular disease risk factor, as well as the value of antihypertensive treatment to reduce cardiovascular events and mortality [3–9]. Automation of BP measurement became favoured over manual methods to lessen the chances of user error from such things as digit preference, observer bias, incorrect stethoscope placement and failing to correctly interpret Korotkoff sounds, to name a few [10, 11]. Consequent efforts were directed towards the development of automated BP measuring devices (BPMDs) based on electronic capture and analysis of pressure waveforms in the cuff, and were specifically designed to provide BP values equivalent to the systolic and diastolic BP values measured with a mercury sphygmomanometer [12].

The first commercial automated BPMD, the Device for Indirect Non-invasive Automatic Mean Arterial Pressure (DINAMAP) 825 [13], became available in 1976 and was incorporated into both research and clinical practice. Appropriately validated automated BPMDs [14] remain the recommended standard for clinical diagnosis and management of hypertension [15–17]. A major reason for the rise in use of automated BPMDs was the global policy directive in 2005 to phase out and replace mercury-based BP measurement in healthcare settings due to environmental toxicity concerns [18, 19]. The two common alternatives to mercury-based BP measurement devices were manual aneroid sphygmomanometers and automated BPMDs [20], with the addition a few years later of the so-called hybrid devices, i.e., manual sphygmomanometers where the mercury column was replaced by a digital led column, associated with an electronic transducer [21]. Preference towards automated BPMDs was widely recommended [22] because of less chance for user error and also because automated BPMDs were perceived to not require the same level of ongoing maintenance required by aneroid devices. However, annual accuracy checking is still advised [23], which is appropriate where resourcing allows, but yet to be proven as a necessary step unless the device has been, or is suspected to be, damaged. It should be noted that calibration of these devices applies to only the pressure transducer. In addition, devices should be regularly inspected for any damage, breaks or tears to the device cuff and tubing, as their integrity is essential to device accuracy. There is now a large global marketplace for automated BPMDs (worth USD 1.5 billion in 2020, projected to reach USD 3.2 billion by 2028) [24] with hundreds of companies manufacturing more than 3500 different models of automated BPMDs, many of which are without evidence of having passed validation testing [25–29].

Despite the ubiquitous availability and use of automated BPMDs, there are few resources available that provide information for the non-specialist audience, not only on how automated BPMDs work, but what they measure compared with invasive and other non-invasive BP reference methods. This paper aims to fill these gaps in the context of this special issue on the accuracy of automated BPMDs [30]. Before describing how automated BPMDs work, it is beneficial to know their operating principles and what is measured by the auscultatory method using a mercury sphygmomanometer because even though this method is phased out of use in most world regions, this is the non-invasive BP reference standard that automated BPMDs were purposefully designed to emulate.

## Auscultatory method using a mercury sphygmomanometer: how does it work, what does it measure?

If we understand the operational strengths and limitations of BP measurement when conducted via both auscultation using a mercury sphygmomanometer and automation using BPMDs, this will enable greater context regarding the performance of the latter, as well as insight on potential areas for improvement. Table 1 summarises the principles of operation using a sphygmomanometer, which firstly involves cuff inflation over the upper arm until the blood flow in the brachial artery is fully occluded. The cuff is then slowly deflated by the operator whilst listening to sounds (auscultation by stethoscope) within the brachial artery at the lower border of the cuff, at the same time as viewing the pressure level within the cuff displayed in millimetres of mercury on the glass column of a sphygmomanometer. Theory states a distinctive sound occurs at the onset of flow under the cuff, with the cuff pressure reading denoting systolic BP (Korotkoff phase I), and the cuff pressure at which sound disappears, or is muffled, denoting diastolic BP (Korotkoff phase V or phase IV, respectively) and occurs with full restitution of blood flow [31]. These brachial artery sounds are separable in time and distinctive from heart sounds [32, 33].

**Table 1:** Summary of the principles of operation for non-invasive estimation of blood pressure (BP) using a manual auscultatory sphygmomanometer compared with automated BP measurement devices (BPMDs).

Operational step	Requirements and rationale for when using a manual auscultatory sphygmomanometer	Comparison with automated BPMDs^a^
Cuff placement	Select an appropriately sized inflatable compression cuff that encircles the upper arm: • Dimensions of the cuff bladder relative to the individual’s mid-arm circumference influence whether proper occlusion of the upper arm and brachial artery occurs • A cuff that is too small (undercuffing) will overestimate BP and a cuff that is too large (overcuffing) will underestimate BP • Cuffs must have an inflatable bladder length covering 75–100% of the mid upper arm circumference and a bladder width covering 37–50% of the mid-arm circumference [59] • The shape of the cuff is also important in large arm circumference where the arm tends to be conical [68]	The 75–100% and 37–50% rule for inflatable bladder dimensions do not apply here. Individualised cuff selection should be based on the mid-arm circumference range indicated on the device cuffs and each cuff available for use with the device must be included in that device’s validation testing
Arm positioned	Arm supported with the middle of the cuff positioned at mid-heart level: • Due to effects of hydrostatic pressure, if the upper arm is above or below the mid-heart level, accuracy of BP readings will be influenced • If the upper arm is too high, BP will be underestimated and if the upper arm is too low, BP will be overestimated (by approximately 0.8 mmHg per cm above or below the heart)	Same requirements and rationale
Cuff inflation	Inflate manually with bulb to at least 30 mmHg above the point where the radial pulse disappears, indicating that the brachial artery is occluded	Same requirement to occlude the brachial artery; however, this is automated in non-hybrid BPMDs. Inflation is controlled electronically by a microcomputer and pumps to a level above systolic BP (e.g., 20–40 mmHg). The level of inflation is determined by proprietary algorithms, with some using stepped cuff pressure changes. Cuff pressure is sensed by pressure transducer. Some devices measure BP during inflation
Cuff deflation	Deflation rate should be 2–3 mmHg/s or per heart rate when heart rate is very slow. A deflation rate that is too fast can significantly underestimate systolic BP and overestimate diastolic BP	Deflation rate is electronically controlled via a deflate valve using a continuous or stepped decrease approach. In most devices the rate of deflation is faster than that recommended for manual measurement for BPMDs that do not use an auscultatory method [69]
Signal	Auscultation by stethoscope placed over the brachial artery in the antecubital fossa below the lower border of the cuff to minimise noise artefact	• For the oscillometric method, the compression cuff and its entrained air volume is used to sense the volumetric changes in the brachial artery created by cardiac contraction and relaxation, resulting in volumetric and therefore pressure changes within the cuff (so-called oscillations). Cuff pressure is sensed by a solid-state pressure transducer within the internal housing of the device • A small number of BPMDs employ an automated auscultator method using a microphone embedded in the cuff with which to detect Korotkoff sounds
Signal association with systolic and diastolic BP	Korotkoff sounds denote systolic BP (phase I) and diastolic BP (phase V, or phase IV in absence of V): • Korotkoff phase I sound is the first appearance of two consecutive clear tapping sounds denoting the introduction of blood flow under the cuff • Korotkoff phase II–IV sounds change in quality as the cuff is deflated • Korotkoff phase V sound is the point at which all sounds disappear, denoting the restoration of blood flow under the cuff	• For the oscillometric method, systolic and diastolic BPs are estimated typically by characteristic ratios of an envelope fitted to the ‘oscillations’ with systolic BP at about 50% (range 45–73%) of maximal amplitude on the rising phase of the waveform envelope and with diastolic BP at about 70% (range 69–83%) of maximal amplitude on the falling phase of the waveform envelope [45, 50] • Mean arterial pressure is estimated on the oscillometric waveform envelope at the point of maximal amplitude • Digital readouts are provided for systolic and diastolic BPs and occasionally for mean arterial pressure • Algorithms for device functionality and those used to estimate mean arterial pressure, systolic and diastolic BP are closely guarded trade secrets that are not shared publicly nor independently scrutinised [45]

^a^Descriptions provided are generally applicable to automated BPMDs but differences exist between manufacturers and devices.

The clinical value of peripheral BP measurement by sphygmomanometer with respect to hypertension is that it gives an estimation of the pressure load experienced by the central organs that are most susceptible to damage from high BP, especially the heart, brain and kidneys [34]. Importantly, the systolic BP at the central aorta level can be significantly amplified as the pressure pulse is transmitted to the brachial artery with each cardiac contraction [35]. The degree of systolic BP amplification varies markedly between individuals, with examples of this variation using invasively measured human data showing little difference (<5 mmHg) between the aorta and brachial artery in some people, but large difference (>30 mmHg) in others [36–39]. On average, brachial artery systolic BP is 8.0 mmHg (95% confidence interval: 5.9 to 10.1 mmHg) higher than that in the proximal aorta, whereas the diastolic BP varies minimally and is only slightly lower at the brachial artery (−1.0 mmHg; 95% confidence interval: −2.0 to −0.1 mmHg) [38]. The wide variability in systolic BP between central and peripheral large arteries naturally raises the question as to what is being measured by a sphygmomanometer at the brachial artery.

As early as in 1951, a committee of the Council for High BP Research of the American Heart Association reported that the sphygmomanometer auscultation method underestimates intra-arterial brachial systolic BP by an average of 3–4 mmHg, but overestimates diastolic BP by an average 8 mmHg [40]. The committee also emphasised the sizable level of scatter, whereby the mean error of the cuff method averaged 8 mmHg from intra-arterial brachial BP for both systolic and diastolic BP [40]. These observations were similar to those of an individual patient level meta-analysis among more than 300 people published in 2017 [38]. The consequent results for pulse pressure measured by sphygmomanometer were underestimation by an average of 11 mmHg, and with a mean absolute difference of 11.8 mmHg (95% confidence interval 9.1 to 14.7) indicating wide scatter from intra-arterial measures [38]. These findings were also replicated in a recent pooled meta-analysis [41].

Altogether, on average, systolic BP measured by auscultation with a sphygmomanometer variably underestimates intra-arterial brachial systolic BP, systematically overestimates diastolic BP and systematically underestimates pulse pressure. The causes of the cuff discrepancies from intra-arterial BP are not fully known, although arterial occlusion itself could create systematic error [42]. The systematic overestimation of diastolic BP probably occurs from incomplete transmission of cuff pressure to the brachial artery, meaning that the arterial segment opens at an intra-arterial pressure that is lower than that exerted by the cuff. The variability in error for systolic and diastolic BP plausibly has interaction with arterial stiffness, resulting in Korotkoff sounds I and V being separate events from the exact movement of the cuff pressure past the systolic and diastolic BP [43].

## Automated BPMDs: how do they work, what do they measure?

**Fig. 1:**
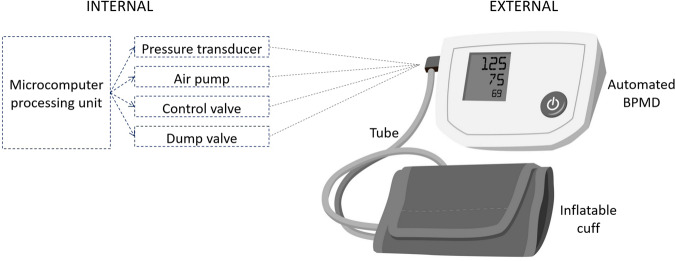
Summary of components for the operation of an automated blood pressure measurement device (BPMD) using the oscillometric method. Cuff inflation and deflation is controlled by a microcomputer with a miniature air pump and valve system to change cuff pressure and facilitate BP measurements. Pressure waveforms generated by cardiac contraction and relaxation are sensed by the inflated cuff and transmitted via the tube to the internal pressure transducer, which provides the input signal for estimation of systolic and diastolic BP via the processing unit.

As detailed in Table 1, automated BPMDs follow the same operational steps as for using manual auscultation with respect to cuff placement and arm position. The need for cuff inflation and deflation also follows the same rationale towards occluding the brachial artery and measuring arterial signals transmitted to the cuff to estimate BP. For automated BPMDs, these processes are undertaken electronically and based on proprietary algorithms designed to estimate BP by analysis of cuff pressure waveform signals detected because they are transmitted from the cuff into the tubing system (and onwards to the pressure transducer), and ultimately processed by microcomputer. This approach has remained mostly unchanged for decades [13, 44, 45]. Key components of automated BPMD measuring systems have been described elsewhere [13, 46] and are summarised in Fig. 1.

Automated BPMDs are traditionally called ‘oscillometric’ BP devices on the basis that the waveforms recorded by cuff and subsequently analysed for BP estimation are oscillometric pulsations. This is a misnomer leading some experts to recommend that automated BPMDs should not be referred to as ‘oscillometric’ devices [47, 48]. Oscillations are periodic waves with a repetitive variation of a measure about a central value, such as an alternating current, whereas the cuff recorded waves are featured brachial artery pressure waves generated from each cardiac cycle [47, 48]. In the spirit of using technically correct terminology, this paper and others in the special issue refer to BPMDs as ‘automated BPMDs’ (this description also includes the small percentage of BPMDs that operate via an automated auscultation method using microphones embedded in the cuff). However, for ease of connection with existing literature on operating principles, the explanations below also refer to conventional oscillometric terms.

Most automated BPMDs analyse the pressure waveform signals during the period of cuff deflation, although some devices analyse signals during the inflation period [49]. The recorded cuff deflation curve has two characteristics: (1) the slowly declining component as cuff pressure is reduced, and (2) the pulsations caused by cardiac contraction and relaxation. The pulsations first become apparent before registration of systolic BP with Korotkoff phase I [12]. The pulsations are extracted and analysed for estimation of BP. The extracted component is referred to as the oscillometric waveform, which is filtered (using various methods) to remove frequency components belonging to the deflating cuff pressure (this filtering step changes the waveform morphology such that classic arterial waveform features may no longer be apparent, and this could be part of the reason contributing to the notion that these are oscillometric waves rather than arterial pressure waveforms). After filtering, an oscillometric waveform envelope is then constructed from the oscillometric waveform using signal processing methods that differ between device makers and also between different models from the same manufacturer, and proprietary algorithms are employed to estimate BPs from the waveform envelope [50].

**Fig. 2:**
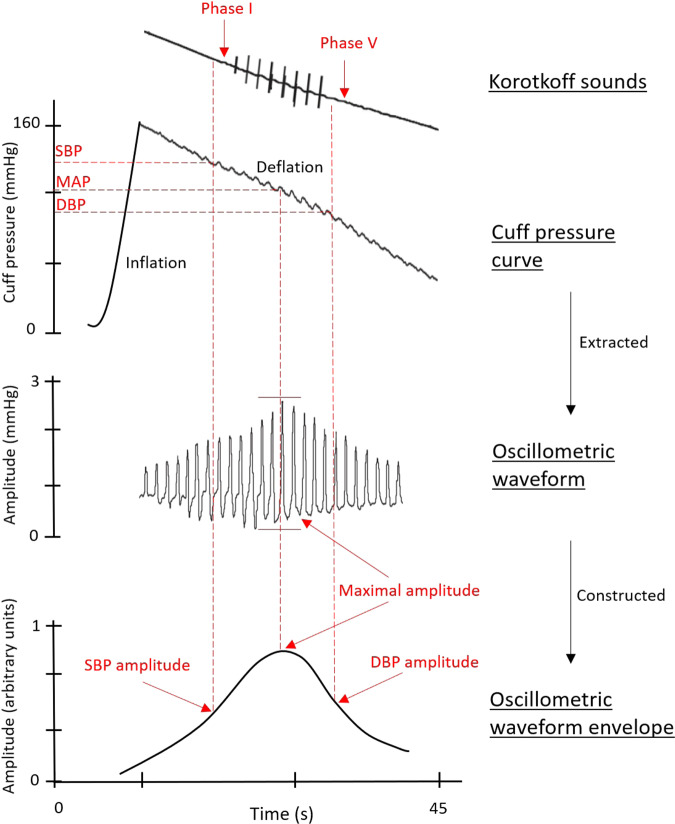
Overview of the oscillometric method to estimate blood pressure (BP) with superimposed example Korotkoff sounds phase I and V. The oscillometric waveform extracted from the cuff pressure curve is processed to construct the oscillometric waveform envelope, whereby the mean arterial pressure (MAP) is identified at the maximal amplitude of the envelope. Systolic BP (SBP) and diastolic BP (DBP) are then estimated using empirical fixed-ratio coefficients or other algorithms.

The maximum amplitude algorithm is a conventional method to estimate mean arterial pressure, which is the cuff pressure at the maximum amplitude of the oscillometric waveform envelope, corresponding with unloading the arterial wall and where the transmural pressure is zero [44, 51, 52]. The systolic and diastolic BPs are estimated using proprietary algorithms, for example, based on empirical fixed-ratio coefficients [50, 53] that are designed to coincide with BPs measured by an auscultation sphygmomanometer. The systolic BP fixed-ratio coefficient correlates with a point on the envelope where the wave amplitude approximates 50% (0.50), and the diastolic BP fixed-ratio coefficient correlates with point where the wave amplitude approximates 70% (0.70) of the maximal amplitude. However, the range of optimal coefficients for accurate systolic and diastolic BP measurements varies greatly between devices [12, 45, 50, 54]. Furthermore, different methods used to construct the oscillometric waveform envelope, as well as individual differences in the shape of the waveform envelope, can lead to different estimated BP values [55, 56]. An overview of the ‘oscillometric’ methods used in automated BPMDs is provided in Fig. 2.

Since each unique automated BPMD can have different processes, algorithms and fitting functions to derive BPs (none of which are publicly disclosed), the accuracy of each BPMD needs to be individually determined by comparison to a BP reference standard using a scientifically accepted validation protocol [14, 57, 58]. This is usually performed non-invasively using an auscultatory sphygmomanometer at the upper arm as the reference, but can also use invasive (intra-arterial catheter) BP monitoring as the reference, especially for devices that are intended for use in the critical care or anaesthetised patient setting [59]. Some limitations of the oscillometric method lead to errors in comparison with manual auscultatory measurement (e.g., up to 10–15%) [60] in BP estimations that are either systematic, random or associated with clinical characteristics of specific patient populations [61].

It must be noted that references to bias, error or accuracy applying to the difference between invasive and non-invasive techniques, do not suggest that an automated BPMD calibrated to auscultatory values should not be used. In fact, the opposite applies. An automated BPMD calibrated to invasive measurements should not be used in an office or home situation where reference clinical values have been obtained non-invasively. Some devices allow the measured value to be switched between non-invasive and invasive.

Among BPMDs using fixed-ratio coefficients, there is systematic bias towards greater underestimation of systolic BP as systolic BP increases, because the optimal fixed-ratio coefficient for accurate systolic BP estimation becomes progressively lower as systolic BP increases (e.g., the optimal fixed-ratio approximates 0.57 at 100 mmHg but this falls to 0.45 at 190 mmHg) [12]. Mechanical properties of the arterial wall can also influence BP accuracy using fixed-ratio coefficients. In particular, increased arterial stiffness leads to overestimation of systolic, diastolic and mean arterial BPs [60, 62–64]. While auscultation is prone to significant errors with fast deflation, automation can be much less prone to these errors, depending on the analysis technique used, as shown by Zheng et al. [65]. However, they studied three repeat measurements, and also showed that variability between repeat measurements almost doubled with fast deflation, but could be improved with oscillometric modelling techniques. Automated devices rely on the oscillatory envelope retaining its shape, and this can be compromised with fast deflation, and slow or variable heart rates. Table 2 summarises several sources of potential error using the oscillometric method. When compared with intra-arterial brachial BP, on average automated BPMDs underestimate systolic BP to a greater degree than with an auscultation sphygmomanometer (−8.0 vs −3.4 mmHg), but the overestimation of intra-arterial diastolic BP is similar (4.5 vs 6.3 mmHg). The underestimation of intra-arterial brachial systolic BP by automated BPMDs means that the estimated systolic BP may be similar to intra-arterial central aortic systolic BP, but this is a device-specific performance characteristic that may vary between individuals [38].

**Table 2:** Potential sources of error from specific components of the oscillometric method to estimate blood pressure (BP).

Oscillometric component	Potential influence on accuracy
Cuff size	A narrow cuff requires higher pressures to induce maximum pulsations, thus increasing the oscillometric waveform to higher pressures for mean arterial pressure, systolic BP and diastolic BP, but also broadening the oscillometric waveform and increasing pulse pressure [61]
Cuff fit	Compared with a cuff that is placed with a snug fit, the estimation of oscillometric mean arterial pressure increases with the looseness of the cuff fit [68]. In large arms, the shape of the cuff is also important to fit the arm that is conical [69]
Cuff deflation rate	Faster deflation rates are used to reduce BP measurement time but this results in fewer oscillometric waveforms with which to estimate BPs. Missing oscillometric waveform values require different degrees of interpolation relative to deflation rate. Although accuracy may not always be compromised [70], error is expected to increase at faster deflation rates [56, 71]
Extracting the oscillometric waveform	Different filtering methods used to extract waveform pulsations can distort the shape of the extracted pulses and the oscillometric waveform, thus changing BP estimations [72, 73]
Constructing the oscillometric waveform envelope	Several signal processing methods can be used to construct the oscillometric waveform envelope, and this creates variability in waveform envelope morphology as well as different BP estimations [55]
BP algorithms	There is a large variety of algorithms to estimate BP, many of which are patent protected for each individual automated BPMD, and have variable performance characteristics [50, 55] that are difficult to independently scrutinise. Algorithms are empirical and may not account for individual physiological variability in modifying factors such as arterial stiffness [74]. Optimal fixed-ratio coefficients for accurate BP also differ depending on the method used to construct the oscillometric waveform envelope [55]

**Fig. 3:**
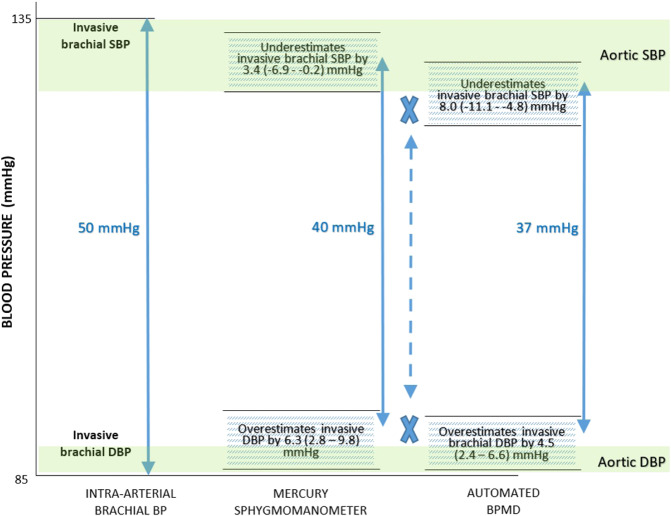
Summary of the level of difference from intra-arterial brachial systolic and diastolic BP for cuff BP measured by sphygmomanometer or automated BP measuring device (BPMD). Data represent mean difference (95% confidence intervals) and are derived from individual participant level meta-analysis in ≥309 participants for BP measured by sphygmomanometer and ≥354 participants by automated BPMDs [38]. Example pulse pressures are denoted by the solid arrows. Green shaded areas represent indicative zones for aortic systolic and diastolic BP. The crosses indicate zones where non-invasive BP is markedly different from either brachial or aortic intra-arterial BPs, and the hatched arrow represents the potential underestimation of pulse pressure using an automated BPMD. Figure modified from [66].

A summary of the level of differences between intra-arterial BP and BP obtained via automated BPMDs and auscultation using a sphygmomanometer is presented in Fig. 3. The figure highlights that divergence from true intra-arterial BP values is greatest for automated BPMDs, and this issue forms a major component of the rationale to develop new BP technology to provide better estimates of BP, especially at the central aortic level [66]. This prospect does not detract from the long-established clinical value of cuff measured BP, and although the general approach of estimating BP by automated BPMDs works well for many people, the method requires refining for improved accuracy among individuals [67]. Whether more accurate non-invasive BP measurement leads to more efficient prevention of cardiovascular disease has been cited as an important question ‘on BP measurement methodology that the scientific community should put on its research agenda’ [68].

## Conclusions

Globally, there are many thousands of unique models of automated ‘oscillometric’ BPMDs available for consumer purchase. Automated devices are also routinely used by healthcare professionals for office/clinic and 24-h ambulatory BP monitoring. This paper has reviewed the operating principles and BP measurement outputs acquired using manual auscultation and automated BPMD methods. The original evidence to support the clinical use of BP measurement was derived with data from the auscultatory sphygmomanometer method using a mercury column, and automated BPMDs were designed to provide equivalent BP values. The automated BPMD method employs a standardised approach that is largely unchanged over many decades, which includes using proprietary empirical algorithms for analysing arterial waveforms and estimating BP. Although there are well-known sources of error associated with automated BPMDs, including variable underestimation when compared with brachial intra-arterial systolic BP, appropriately validated BPMDs provide similar values to auscultatory BP, albeit with room for improved accuracy that is expected to refine individual risk stratification for better cardiovascular disease risk prevention and improved treatment and management of hypertension.
